# 

**Published:** 1864-09

**Authors:** 


### BOOKS AND PAMPHLETS RECEIVED.

*Military Medical and Surgical Essays prepared for the United States Sanitary Commission. Edited by* WILLIAM A. HAMMOND, M. D., *Surgeon-General United States Army, etc. Philadelphia:* J. B. LIPPINCOTT & Co., 1864.

*A Treatise on Gonorrhœa and Syphilis.* BY SILAS DURKEE, M. D., *consulting Surgeon of the Boston City Hospital; Fellow of the Massachusetts Medical Society; Member of the Boston Society for Medical Improvement; Honorary Member of the Medical Society of the State of New York; Fellow of the American Academy of Arts and Sciences, etc. Second edition, revised and enlarged, with eight colored illustrations. Philadelphia:* LINDSAY & BLAKISTON, 1864.

*The Physician's Dose and Symptom Book, containing the doses and uses of all the principal articles of the Materia Medica and Officinal preparations; also the Tables of Weights and Measures, Rules to Proportion the Doses of Medicine, Common Abbreviations used in writing Prescriptions, Table of Poisons and Antidotes, Classification of the Materia Medica, Pharmaceutical Arrangement, Table of Symptomatology, Outlines of General Pathology and Therapeutics.* BY JOSEPH H. WYTHES, A. M., M. D., *author of "The Microscopist," "Curiosities of the Microscope," etc., etc. Fourth edition. Philadelphia:* LINDSAY & BLAKISTON, 1864.

*The Physician's Visiting Diary and Book of Engagements, for* 1865. *Philadelphia:* LINDSAY & BLAKISTON.

*Twenty-Second Annual Announcement of Rush Medical College, Chicago, Illinois, for the Session of* 1864–5, *with Catalogue of previous session.*

*Sixth Annual Announcement of the Chicago Medical College, Medical Department of Lind University, Chicago, Illinois, for the College Session of* 1864–5.

